# PReS-FINAL-2127: Sclerodermatous graft versus host disease

**DOI:** 10.1186/1546-0096-11-S2-P139

**Published:** 2013-12-05

**Authors:** JR Stevenson, C Mcvitty, N Martin, B Gibson, AM Ewins, L Jones, D Shanks, C Grant, M Berry, J Duncan, JE Davidson

**Affiliations:** 1 Yorkhill RHSC, Glasgow & Clyde, Glasgow, UK; 2 NHS Greater Glasgow & Clyde, Glasgow, UK; 3 RHSC, Edinburgh, NHS Lothian, Edinburgh, UK; 4 Raigmore Hospital, Inverness, NHS Highland, Inverness, UK

## Introduction

Cutaneous graft-versus-host-disease (GVHD) is a common manifestation of GVHD post allogeneic haematopoietic stem cell transplantation (HSCT). It is characterised into lichenoid and scleroderma variants. Sclerodermatous GVHD is thought to be rare, with few reports in the paediatric population.

## Objectives

To describe 3 paediatric cases of GVHD associated scleroderma.

## Methods

Retrospective case note review.

## Results

A 7 year old girl was referred to rheumatology 2 years after maternal HLA matched HSCT for acute lymphoblastic leukaemia. Mild cutaneous GVHD was noted post transplant. She subsequently presented with severe sclerodermatous changes on both lower legs. She had marked loss of range of movement (ROM) of her ankles, and the disease rapidly progressed to involve the skin of her thighs and left arm, with significant joint contractures. She was treated with methylprednisolone, methotrexate and physiotherapy (PT) with good effect: improved ROM, softening of the skin and no further progression.

A 4 year old girl was referred to rheumatology 3 years after having 2 matched sibling HSCT for MHC class 2 deficiency. Very mild cutaneous GVHD had been noted post transplant. She presented with rapidly worsening contractures affecting her hands and lower limbs with diffuse swelling, erythema, thickness and tethering of skin and tendons. She was treated with PT, occupational therapy (OT) and splinting, together with prednisolone and methotrexate. Clinical improvement was maintained over the following year.

A 6 year old girl with beta thalassaemia major was referred to rheumatology 2 years post HSCT. Buccal GVHD was noted 4 months post transplant, treated initially with steroids and ciclosporin followed by extra-corporeal photophoresis. Despite some initial response to this there was a profound deterioration of hand function with severely restricted wrists and fingers bilaterally and reduced grip strength. She has been treated with an increased dose of prednisolone, the addition of methotrexate, PT and OT.

## Discussion

We report 3 cases of sclerodermatous GVHD following HSCT. The indications for HSCT differed in each case. All had mild cutaneous GVHD noted in the early post transplant period. All presented months or years later with extensive sclerodermatous skin changes. Widespread established joint contractures were noted at the time of referral to rheumatology. There is a paucity of evidence regarding effective treatment for this condition. 2 of our cases showed significant improvement with a combination of steroids, methotrexate, PT, OT and appropriate splinting.

## Conclusion

Sclerodermatous GVHD is rare in the paediatric population but results in significant morbidity in affected individuals. In these cases severe joint contractures were noted at the time of referral to Rheumatology. Monitoring for early signs of joint contracture post HSCT, and raising awareness of this potential complication, should facilitate earlier diagnosis, with the potential to optimise outcome for affected children.

## Disclosure of interest

None declared.

